# Investigating the Clinical Merit of Contraction Patterns for Glabellar Neuromodulator Injections—A Clinical Retrospective Investigation Following the 3‐Point Glabellar Injection Technique

**DOI:** 10.1111/jocd.70147

**Published:** 2025-04-12

**Authors:** Marcelo Germani, Victor R. M. Munoz‐Lora, Jeremy B. Green, Brain S. Biesman, Galen Perdikis, Rod J. Rohrich, Arthur Swift, Michael Alfertshofer, Sebastian Cotofana

**Affiliations:** ^1^ Department of Biological Sciences, Bauru School of Dentistry University of São Paulo Bauru Brazil; ^2^ Department of Periodontology and Implantology Guarulhos University São Paulo Brazil; ^3^ Skin Associates of South Florida and Skin Research Institute Coral Gables Florida USA; ^4^ Department of Plastic Surgery Vanderbilt University Medical Centre Nashville Tennessee USA; ^5^ Private Practice Dallas Texas USA; ^6^ Private Practice Montreal Canada; ^7^ Department of Oromaxillofacial Surgery Ludwig‐Maximilians‐University Munich Munich Germany; ^8^ Department of Dermatology Erasmus University Medical Center Rotterdam the Netherlands; ^9^ Centre for Cutaneous Research, Blizard Institute, Queen Mary University of London London UK; ^10^ Department of Plastic and Reconstructive Surgery Guangdong Second Provincial General Hospital Guangzhou China

**Keywords:** corrugator supercilii muscle, glabellar contraction patterns, glabellar severity scale, neuromodulator injections, procerus muscle

## Abstract

**Background:**

Since the introduction of glabellar contraction patterns as a guide for neuromodulator treatments, feedback regarding its clinical utility has been mixed. Recent studies have cast doubt on the value of such contraction‐pattern‐driven treatment paradigms.

**Objective:**

The aim of this retrospective study is to evaluate the clinical outcome of identical glabellar injections independent of glabellar contraction pattern types.

**Methods:**

Forty‐two Brazilian, multi‐ethnic, consecutive patients with moderate to very severe glabellar lines were included in this retrospective analysis. The same 3‐point glabellar injection technique was utilized, administering a total of 37.5 sU (=15 IU) to the procerus and corrugator supercilii muscles. Glabellar severity was assessed at 20 and 90 days after the initial treatment.

**Results:**

Across the entire study sample, a highly statistically significant improvement was observed at all investigated follow‐up time points (*p* < 0.001). When comparing the treatment outcome across the five different glabellar contraction patterns, there was no statistically significant difference detected at either 20 days follow‐up (*p* = 0.373) or 90 days follow‐up (*p* = 0.311). Multivariate analyses confirmed that neither age, BMI, Fitzpatrick skin type, nor glabellar contraction patterns statistically influenced the outcome at 90 days (all *p* > 0.05).

**Conclusion:**

The results of this retrospective analysis revealed that in the sample investigated, no statistically significant differences were detected between patients with different glabellar contraction patterns at any of the conducted follow‐up visits when the same glabellar injection technique with the same amount of toxin units was administered.

## 1 Introduction

Neuromodulator injections for reducing facial rhytids remain the most frequently performed minimally invasive aesthetic procedure according to the 2023 annual statistics of the American Society of Plastic Surgeons [1] with close to 9.5 Mio procedures performed in that year. Of the various facial and non‐facial regions that can be targeted with neuromodulators [2, 3, 4], the upper face is the most frequently requested facial region, with special emphasis being placed on forehead and glabella rhytids [5].

In 2010 and 2012, de Almeida et al. [6, 7] presented their concept of glabellar contraction patterns, which suggested modification of the FDA‐approved injection paradigm according to certain skin wrinkle types. The authors recommended employing different injection points depending on which glabellar contraction type was present. Subsequently, various authors published about their inconsistent results with this suggested injection algorithm in Korean, Indian, and Chinese patient populations [8, 9, 10, 11].

In 2024, Rams et al. [12] tried to identify differences in morphologic features of glabellar muscular anatomy by utilizing magnetic resonance imaging in young, healthy, toxin‐naïve individuals. The authors found no statistically significant differences in muscle length, width, and thickness of the glabellar muscles, including procerus, corrugator supercilii, orbicularis oculi, and frontalis muscle when stratifying their sample into glabellar contraction patterns according to de Almeida et al. [6, 7] Their results implied that, independent of which contraction pattern was present, the evaluated muscle parameters were similar across the investigated study sample.

However, the study by Rams et al. [12] was an imaging‐based study without a clinical arm in which neuromodulator injections were conducted, leaving that study without foundational clinical validity. To better evaluate the possible clinical merit of utilizing glabellar contraction patterns when targeting the glabella with neuromodulators, the current clinical study was designed. The aim of this study was to compare the outcome of glabellar neuromodulator injections between patients with different glabellar contraction types and to assess whether the off‐label modification initially suggested by de Almeida et al. when treating glabellar rhytids with neuromodulators was necessary.

## 2 Materials and Methods

### 2.1 Study Design

This study was designed as a retrospective analysis of previously conducted glabellar injections administered between February 2024 and November 2024, and it received ethical approval from the Research Ethics Committee of the “Centro Universitário Católico Salesiano Auxilium” under the protocol number CAAE—70079723.1.0000.5379. All patients provided written informed consent prior to their inclusion for the use of their demographic and clinical data for medical research purposes. The initial treatments conducted (glabellar injections with neuromodulators) adhere to the departmental standards of care and to the guidelines of Good Clinical Practice.

### 2.2 Study Sample

Forty‐two (*n* = 42) consecutive, Brazilian, multi‐ethnic, previously treated patients with moderate to very severe (Table 1) glabellar lines were included in this retrospective analysis. No specific inclusion criteria were applied to allow for a community‐based study approach. Exclusion criteria included individuals with pre‐existing medical conditions such as neuromuscular disorders, blood clotting issues, known allergies to neuromodulators or their components, active infections in the treatment area, pregnant or breastfeeding women, patients who had undergone facial cosmetic surgeries, as well as those who had received any form of aesthetic treatment, including neuromodulators, in the 12 months prior to receiving their glabellar neuromodulator treatments. The primary outcome of this study was the evaluation of the investigated parameters at 90 days follow‐up; the 20 days follow‐up was used as an intermediate data evaluation point.

**TABLE 1:** Demographic description of the study sample investigated. Across group differences (glabellar contraction pattern groups) were computed with One‐way ANOVA for age and body mass index (BMI) and with Friedman test for glabella severity (GLSS).

Demographics	Value
Participants	42
Male	5
Female	37
Age	41.3 (±9.18)
BMI	24.50 (±4.73)
GLSS	2.00 (IQR = 1.00)
Group V‐shape	6
Age	37.8 (±4.96)
BMI	23.3 (±5.64)
GLSS	2.00 (IQR = 0.00)
Group U‐shape	15
Age	43.1 (±9.18)
BMI	26.5 (±9.6)
GLSS	2.00 (IQR = 0.00)
Group converging arrows	14
Age	41.3 (±10.80)
BMI	24.3 (±4.91)
GLSS	3.00 (IQR = 1.00)
Inverted omega	4
Age	46.3 (±4.79)
BMI	24.2 (±9.6)
GLSS	1.50 (IQR = 1.50)
Omega	3
BMI	32 (±6.24)
Age	21.3 (±3.83)
GLSS	2.00 (IQR = 0.00)

*Note:* No statistically significant differences were found in any of the conducted tests. Results are presented as frequency count for sex distribution, as mean and 1x standard deviation for age and BMI, and as median and the respective interquartile range (IQR) for GLSS.

### 2.3 Glabellar Contraction Pattern

**FIGURE 1:**
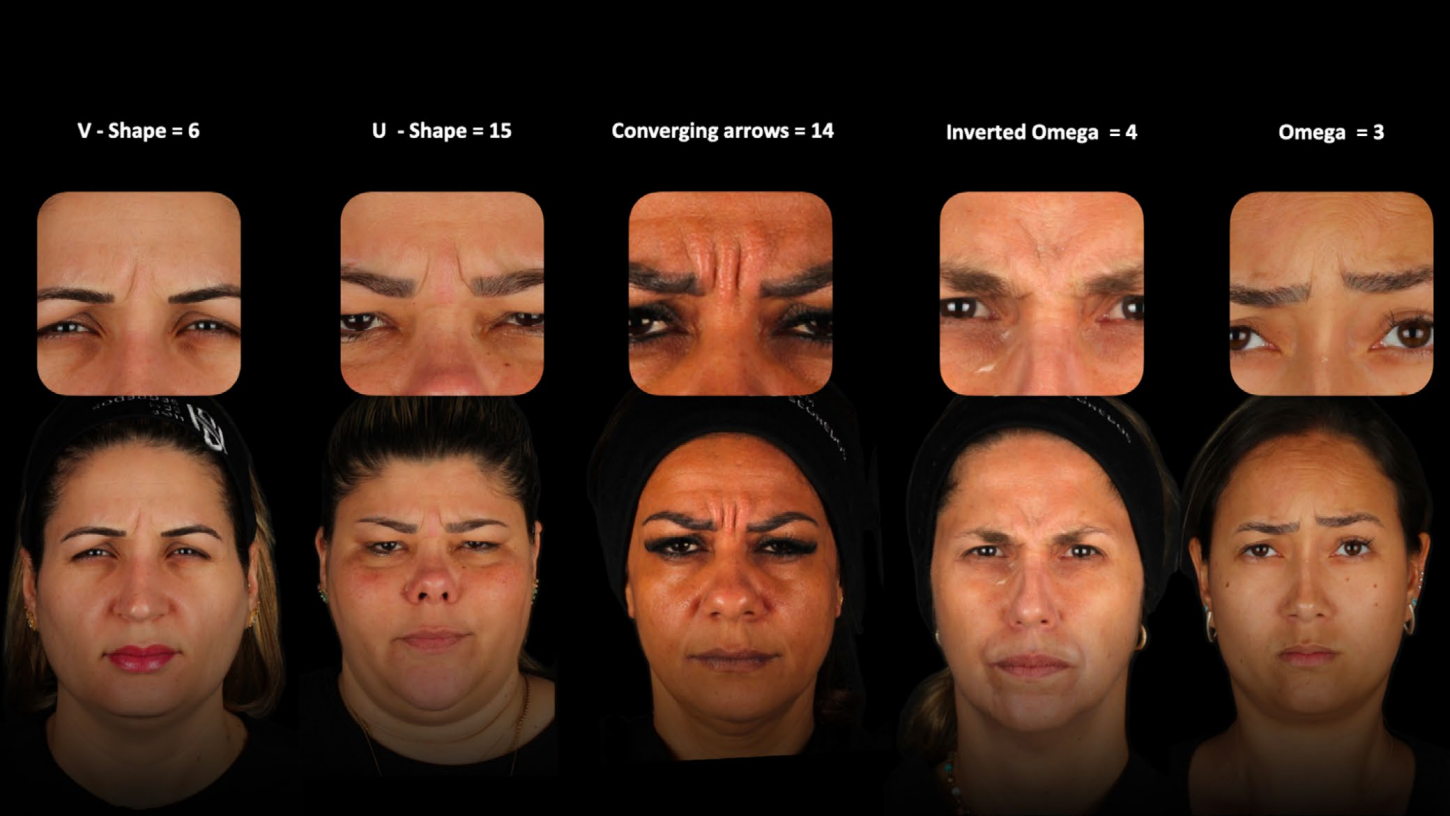
Study participants classified according to the previously published five glabella contraction patterns [6, 7].

Patients were instructed to frown as strongly as possible, activating the glabellar muscles (procerus [PM], corrugator supercilii [CSM], orbicularis oculi [OOM], and frontalis [FM]). The resulting wrinkle patterns on the skin surface were classified into categories based on skin surface rhytid patterns described previously by de Almeida et al. [6, 7] U‐shape, V‐shape, Converging Arrows, Omega, and Inverted Omega (Figure 1).

The classification was conducted via 2‐dimensional facial images by two independent evaluators, each with at least 5 years of experience in facial aesthetics. In cases where there was no consensus between the evaluators, a third evaluator was consulted to finalize the classification. This ensured consistency and reliability in the categorization of the glabellar contraction pattern.

### 2.4 Injection Technique

All neuromodulator injections were performed in a single session by a single practitioner (M.G.) with over 5 years of experience in facial toxin treatments. The product utilized in all treatments was abobotulinumtoxinA (Dysport, Galderma, Uppsala, Sweden) and was always prepared the same day of treatment with 2.0 cc of sterile saline solution (Samtec, Ribeirão Preto, Brazil) added to each 500 sU (= 200 IU) vial. All treated areas were cleaned with 2% chlorhexidine, and topical anesthesia (4%, Dermomax, Aché Laboratorios Farmacêuticos, Guarulhos, Brazil) was applied to the designated injection sites. Injections were administered using a 31G/6 mm syringe (Becton Dickinson, Franklin Lakes, NJ, US).

**FIGURE 2:**
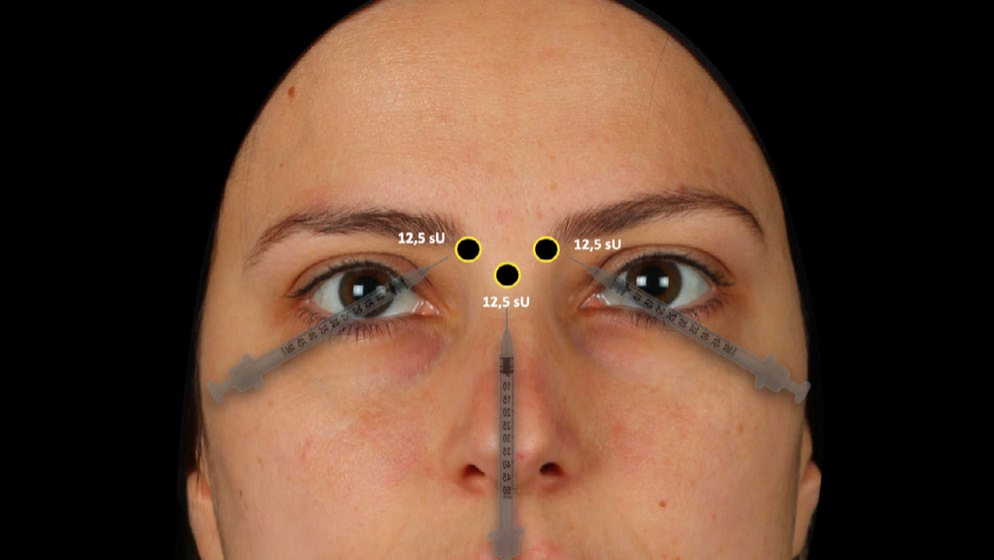
Representation of the conducted injection technique. sU = Speywood units.

All patients were treated equally using a 3‐point injection technique as previously described by Cotofana et al. in 2021 [13]. They did not receive any other treatment of their face with neuromodulators. For the corrugator supercilii muscles, a single 12.5 sU (= 5 IU) injection was performed at the medial and lower margin of the eyebrows, with the needle inserted at a 45° angle toward the midline, establishing bone contact [14]. For the procerus muscle, a deep midline injection with 12.5 sU (= 5 IU) was performed at a horizontal level of the medial canthal ligaments in a 90° angle, establishing again bone contact. The total dose per patient was 37.5 sU (= 15 IU) and was equal for each patient and, importantly, independent of their glabellar contraction pattern. (Figure 2).

The institution (in which all treatments were carried out) most frequently uses the 3‐point glabellar injection technique, and this investigation evaluated patients solely treated with this specific injection algorithm. The study site also utilizes other injection algorithms like the standard 5‐point glabellar technique; however, patients treated with this injection technique were not included in this analysis.

### 2.5 Glabellar Line Severity Scale (GLSS)

The severity of glabellar lines during maximum contraction was assessed before, 20, and 90 days after the treatment using the previously published Glabellar Line Severity Scale (GLSS) [15]. The severity of the glabellar wrinkles was evaluated by two independent, experienced evaluators each with more than 5 years of experience in facial aesthetics on 2‐dimensional facial frontal photographs using a 0 to 4 scale, where 0 = “no lines,” 1 = “mild lines,” 2 = “moderate lines,” 3 = “severe lines,” and 4 = “very severe lines.” In cases where there was no agreement between the two evaluators, a third evaluator was consulted to finalize the rating.

### 2.6 Statistical Analysis

Statistical analyses were conducted using the open source Jamovi software (The Jamovi Project, version 2.3.28, Sydney, Australia), with the significance level set at *p* ≤ 0.05. To ensure a comprehensive understanding of the data, quantitative variables are presented as a mean and 1x standard deviation (SD), while ordinal (categorical) variables are presented as a median and interquartile ranges (IQR). Across group comparisons (glabellar contraction patterns) were conducted either via one‐way ANOVA (parametric testing) or via Friedman test (non‐parametric testing). Within group comparisons (treatment time points; baseline, 20 days, 90 days) were conducted via Kruskal Wallis test (non‐parametric testing). Multivariate ordinal logistic regression models were run to investigate factors influencing the treatment outcome at 90 days post‐treatment when using the GLSS with the inclusion of age, BMI, Fitzpatrick skin type, and glabellar contraction patterns.

## 3 Results

### 3.1 Demographics

This retrospective study included 42 consecutive patients (5 male and 37 female) of Brazilian multi‐ethnic background with a mean age of 41.3 (9.18) years and a mean BMI of 24.5 (4.73) kg/m^2^ at baseline. No statistically significant differences for age (*p* = 0.075) or BMI (*p* = 0.401) were detected when comparing individuals across the five different glabellar contraction pattern groups.

The median GLSS at baseline for the entire study sample was 2.0 (1.0) with no statistically significant differences across the five different glabellar contraction pattern groups (*p* = 0.160). For detailed information regarding the study sample see Table 1.

### 3.2 Glabellar Severity Following the Treatment

**FIGURE 3:**
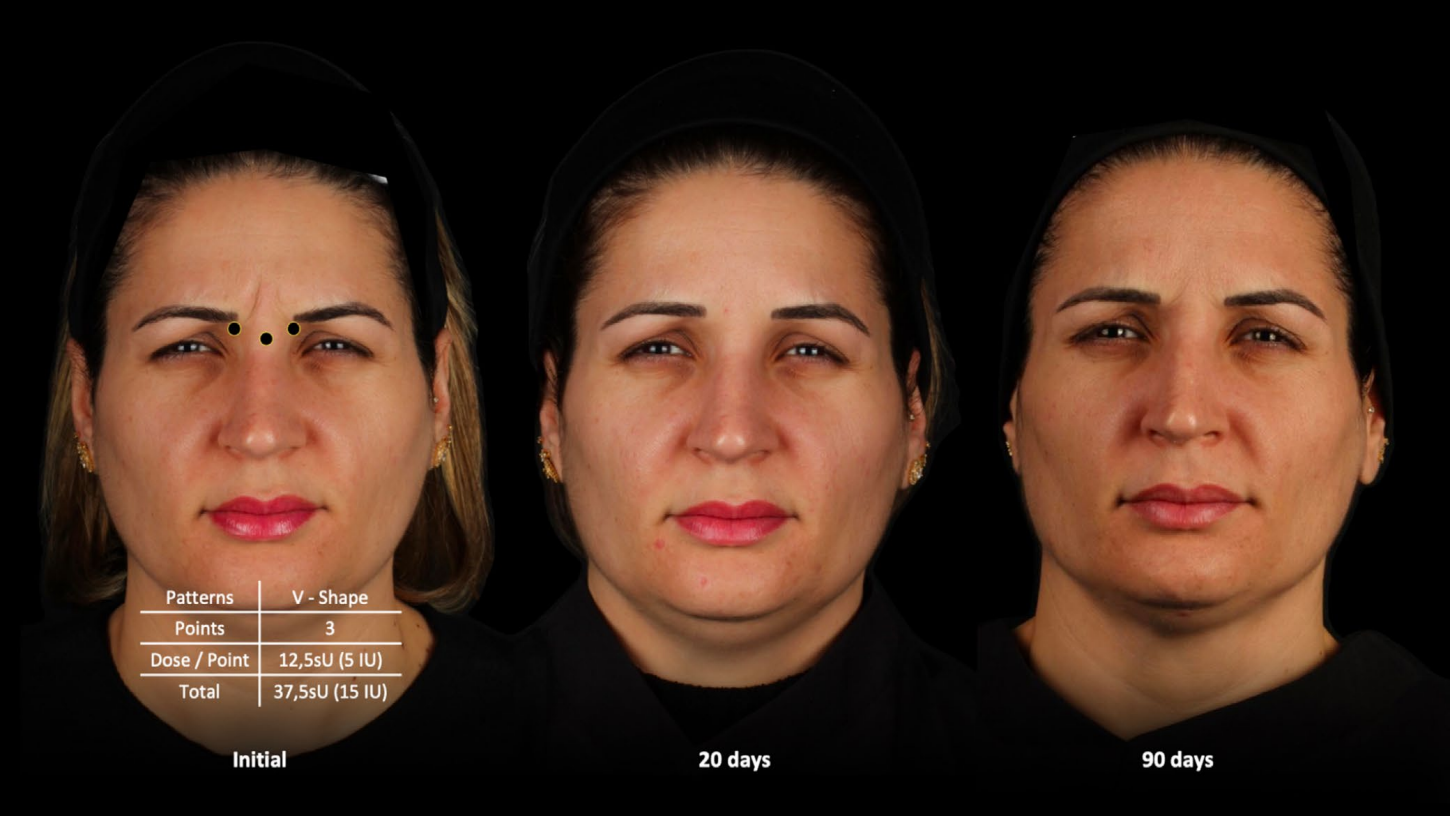
Treatment sequence of a 43‐year‐old female patient treated with a total dose of 37.5 sU (15 IU) of abobotulinumtoxinA. Follow up occurred before, 20 and 90 days after neuromodulator treatment.

**FIGURE 4:**
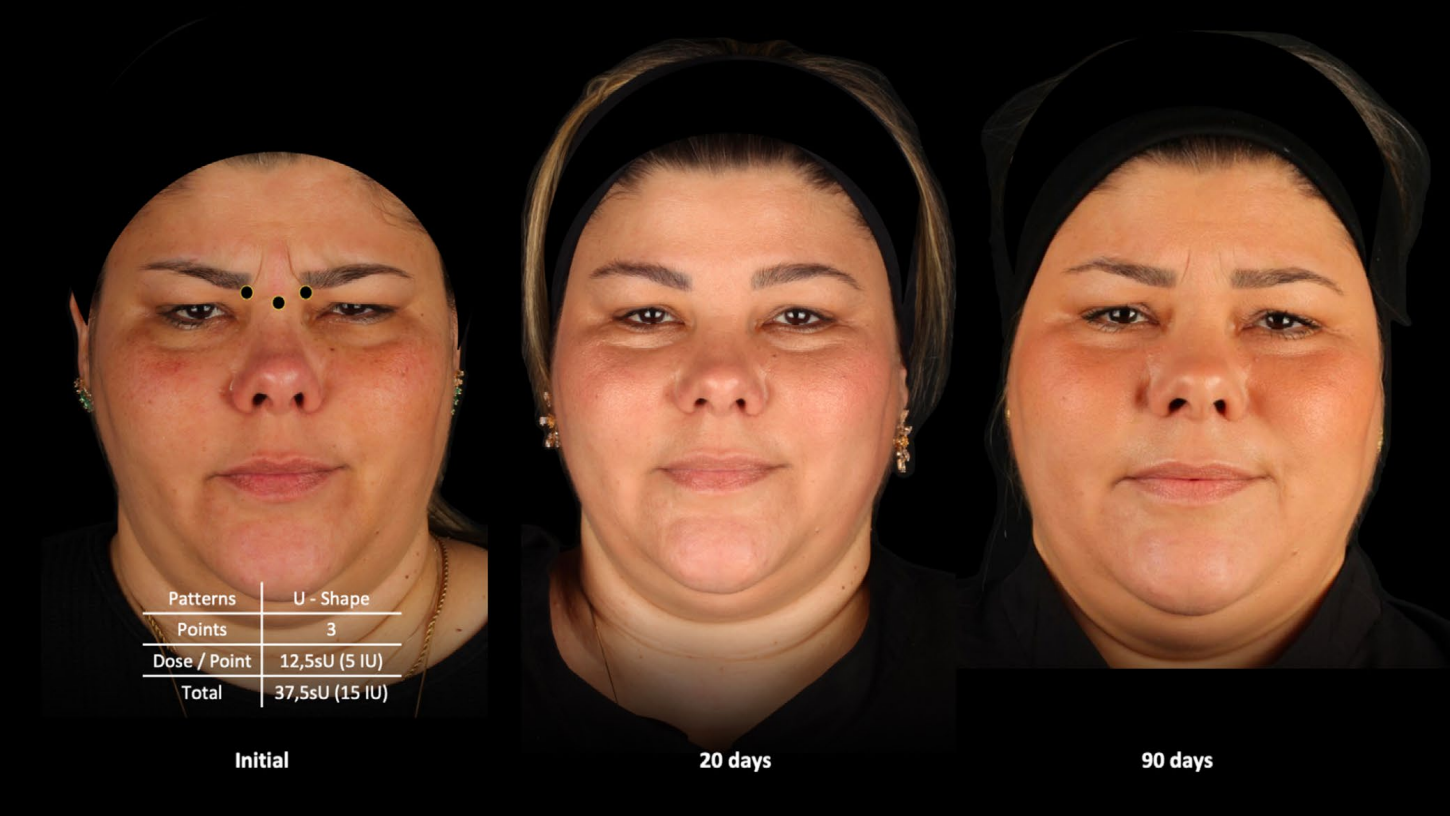
Treatment sequence of a 46‐year‐old female patient treated with a total dose of 37.5 sU (15 IU) of abobotulinumtoxinA. Follow up occurred before, 20 and 90 days after neuromodulator treatment.

**FIGURE 5:**
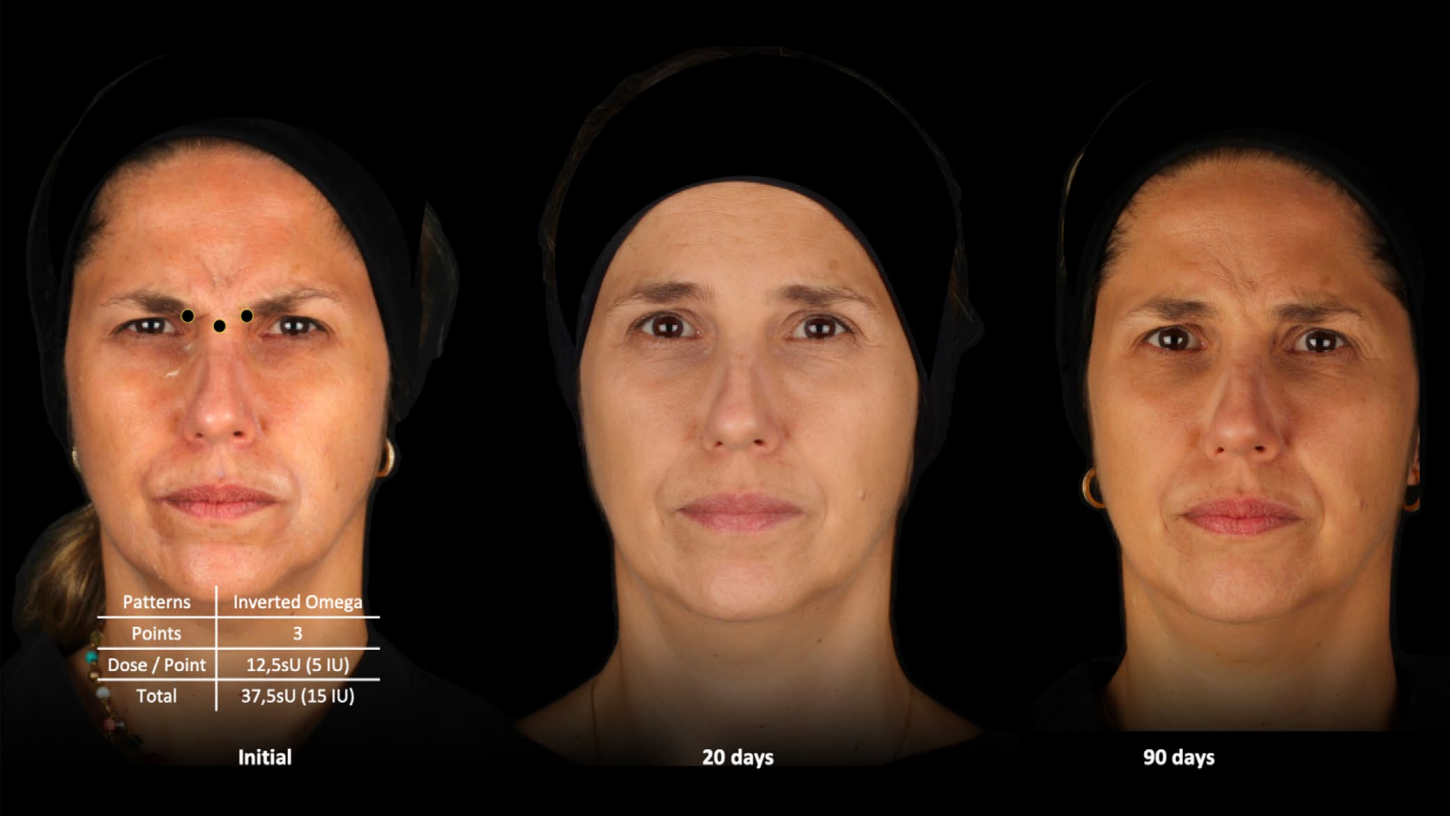
Treatment sequence of a 42‐year‐old female patient treated with a total dose of 37.5 sU (15 IU) of abobotulinumtoxinA. Follow up occurred before, 20 and 90 days after neuromodulator treatment.

The median GLSS 20 days after the initial treatment for the entire study population was 0.00 (0.00) with a median difference of 2.0 when compared to baseline (*p* < 0.001). The median GLSS at 90 days was 1.0 (1.00) with a median difference of 1.0 when compared to baseline (*p* < 0.001). (Figures 3, 4, 5).

**FIGURE 6:**
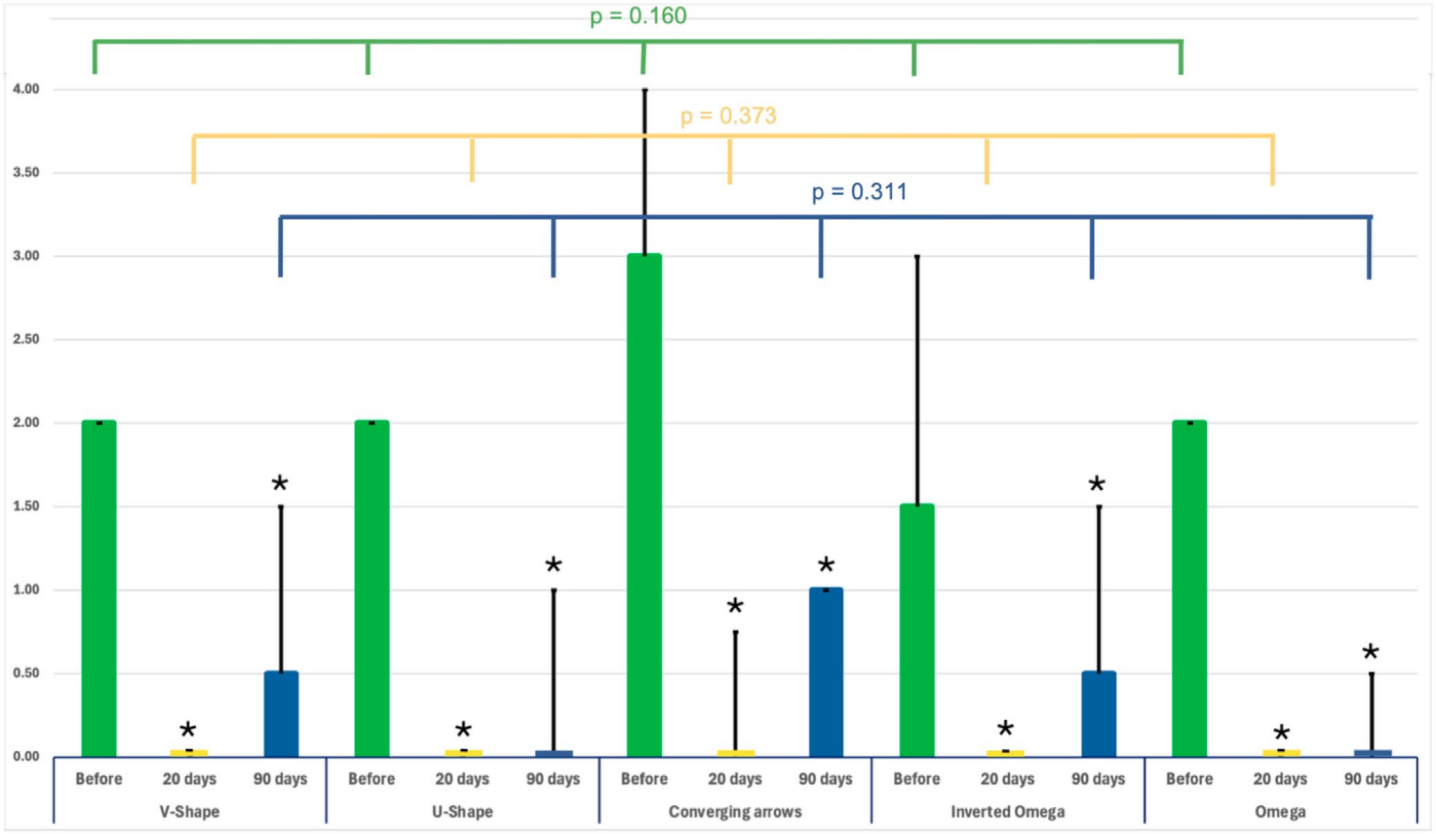
Bar graphs showing the median and interquartile range (IQR) of the glabella contraction patterns before, 20 days and 90 days post‐treatment, stratified by the investigated glabellar contraction patterns. *p*‐values indicate across group differences according to Friedman test. Asterisks indicates statistically significant change when compared to baseline glabellar severity (GLSS) when computed by Kruskal Wallis test.

When comparing the treatment outcomes across the five different glabellar contraction pattern groups, it was revealed that no statistically significant differences were noted at 20 days post‐treatment (*p* = 0.373) or at 90 days post‐treatment (*p* = 0.311) (Figure 6).

### 3.3 Multivariate Analyses of Treatment Outcome

Ordinal logistic regression analyses were conducted to explore factors influencing the GLSS at 90 days post‐treatment. The predictors included age, BMI, Fitzpatrick skin type, and glabellar contraction pattern.

Age and BMI did not impact the likelihood of achieving a lower GLSS at 90 days (*p* = 0.763 and *p* = 0.510, respectively). When comparing Fitzpatrick skin types to the baseline Fitzpatrick Type I, there were no statistically significant differences in treatment outcomes at 90 days (type II: *p* = 0.960, type III: *p* = 0.914, and type IV: *p* = 0.458; all vs. type I).

When investigating the influence of each individual glabellar contraction pattern type (compared to V‐shape used as reference) on the treatment outcome at 90 days, again, no statistically significant differences were noted (U‐shape: *p* = 0.976, Converging Arrows: *p* = 0.135, Inverted Omega: *p* = 0.799, and Omega: *p* = 0.874; V‐shape was used as reference).

### 3.4 Adverse Events

No adverse events were observed during the study observational period (90 days) that could be related to the neuromodulator treatment like medial eyebrow ptosis, upper eyelid ptosis, or lateral hyper‐elevation (“Spock eyebrow”). No follow‐up injections were requested by the patients or deemed necessary by the initial treatment provider.

## 4 Discussion

This study was designed to compare the clinical outcome following the 3‐point glabellar injection technique with neuromodulators between groups of patients with different glabellar contraction patterns. The results of this retrospective analysis revealed that in the sample investigated, no statistically significant differences were detected either at the 20‐day or at the 90‐day follow‐up visit when assessed via the GLSS. These results are not surprising in the context of the recently published work by Rams et al. [12] which utilized MR imaging to compare morphologic features like length, width, and thickness of the glabellar muscles. In that study, the authors found no statistically significant difference when stratifying their sample into the five glabellar contraction patterns described by de Almeida et al. [6, 7], indicating that the underlying morphology of procerus, corrugator supercilii, orbicularis oculi, and frontalis muscles is not different even if the overlying soft tissues present varying skin rhytid patterns with frowning. The authors of that study concluded that neuromodulator injections can be performed with consistently satisfactory clinical outcomes without customizing treatments to glabellar contraction type.

The present study aims to continue the work of Rams and his colleagues by providing clinical support for the findings obtained from MR imaging. The results showed that independent of their glabellar contraction type, patients improved their GLSS at the 20‐day and 90‐day follow‐up visits on a highly statistically significant level with *p* < 0.001 for all 42 investigated subjects. Despite utilizing the same 3‐point glabellar injection technique as published by Cotofana and his colleagues [13] and without adjusting for the different glabellar rhytid patterns, the results revealed a highly statistically significant clinical improvement. It could be, however, argued that in the present study differences other than glabellar contraction patterns might have influenced the clinical outcome.

To investigate this aspect, additional statistical models were computed. It was identified that at baseline, no statistically meaningful difference was present between the investigated contraction patterns when focusing on age or BMI (all *p* > 0.05). When additionally adjusting for age, BMI, and Fitzpatrick skin type in a multivariate model, it was identified that none of the evaluated factors influenced the clinical outcome, with all *p* > 0.05. This finding suggests that glabellar rhytids can be treated with the same standard injection technique (here the 3‐point injection technique) independent of age, BMI, skin type, or glabellar contraction pattern and achieve the same good clinical outcome. This result is consistent with a previous survey‐based study by Cotofana et al. [16] which asked 386 health care providers from 61 countries to rate the facial region that has the greatest difficulty in achieving the perfect aesthetic outcome (if such outcome exists) when administering neuromodulator treatments. The respondents stated that the glabella was the easiest facial area to treat with neuromodulators and achieve a good aesthetic outcome; in contrast, the forehead was rated among the 3 most difficult facial regions. This survey outcome provides additional clinical support for the findings of this retrospective clinical study, which likewise identified that independent of age, BMI, skin type, or glabellar contraction pattern, the glabella can be treated with the same injection algorithm and achieve a good clinical outcome.

However, the question remains as to why the skin forms different rhytid patterns on the surface despite the same underlying morphology of the glabellar muscles. A potential explanation for this interesting anatomic aspect was provided recently by Alfertshofer and his colleagues [17] when they described a biomechanical unit comprised within the upper facial soft tissues consisting of facial muscles, connective tissue envelope, and skin. The authors argued that each of these components can age differently and can therefore present their effects differently on the skin surface upon movement. It could be assumed that between glabellar muscles and the overlying skin a 3‐dimensional apparatus exists which allows for the transmission of contractile movements between muscle fibers and dermal undersurface. The varying composition of this apparatus may therefore contribute to the different skin surface glabellar contraction patterns despite constant underlying glabellar muscle anatomy.

Understanding that neuromodulators have their predominant effect at the neuromuscular junction of facial muscles [18, 19] it seems plausible to employ an injection algorithm that respects the underlying anatomy rather than a skin surface rhytid pattern. Adjusting a neuromodulator injection algorithm to a skin surface pattern and not following the underlying muscular glabellar could potentially result in adverse events like eyebrow ptosis, upper eyelid ptosis, lateral eyebrow hyper elevation, or in suboptimal outcomes regarding efficacy and duration. To avoid the latter effects, it is proposed to avoid making treatment modifications based on glabellar rhytid patterns [6, 7] and employ an anatomy‐ and evidence‐driven injection algorithm.

The present study is not without limitations: First, a more heterogeneous study population would have provided wider generalizability. Second, a larger sample size would have supported a more robust statistical analysis, but given that the present study was conducted as a retrospective and not as a prospective clinical trial, the study sample and the respective sample size are limited in its dimensions a priori. Third, this study utilized the 3‐point injection technique and not the standard 5‐point injection technique for treating the glabella. The reason why this specific injection technique was chosen was due to a previous publication by Cotofana et al. [13] which utilized a 3‐point injection technique to address glabellar frown lines instead of the standard 5‐point injection technique. In that study, the authors utilized 5 I.U. for the procerus muscle but used 13 I.U. to treat each corrugator supercilii muscle. In alignment with that study, we used likewise 5 I.U. for the procerus muscle. But due to the risk of causing upper eyelid ptosis, we reduced the dose for each corrugator supercilii muscle to 5 I.U. and hyper‐concentrated the product with only 2.0 cc for the 200 I.U. vile. This was specifically decided to reduce the risk of product spread toward the levator palpebrae superioris muscle and to avoid treatment‐related upper eyelid ptosis; this is supported by the fact that no adverse events were observed during the study period. Fourth, this study investigated the clinical outcome following the 3‐point injection technique and not the duration of the clinical effect following the injection of 15 I.U. of neuromodulator product. Future studies should investigate the clinical longevity of the 3‐point technique when the dose is escalated to 60 I.U. or more. It must be emphasized that an additional clinical study is needed in which the 5‐point glabellar injection algorithm is conducted, and the clinical outcome is compared across the five different glabellar contraction patterns.

## 5 Conclusion

The results of this retrospective analysis revealed that in the sample investigated, no statistically significant differences were detected between patients with different glabellar contraction patterns at any of the conducted follow‐up visits when the same glabellar injection technique with the same amount of toxin units was administered. Following the results obtained, it is proposed to avoid modifications in glabellar frown line treatments based on skin surface contraction pattern and instead utilize an anatomy‐ and evidence‐driven injection algorithm when addressing glabellar rhytids.

## Author Contributions

All authors significantly contributed to the conception, design, and execution of this study. They participated in data collection, analysis, and interpretation, as well as in drafting and critically revising the manuscript. Each author has reviewed and approved the final version of the article and agrees to be accountable for all aspects of the work, ensuring its integrity and accuracy.

## Conflicts of Interest

The authors declare no conflicts of interest.

## Data Availability

The data that support the findings of this study are available from the corresponding author upon reasonable request.
